# Indoleamine 2,3-dioxygenase-regulated macrophages metabolic reprogramming rescues tacrolimus-induced nephrotoxicity

**DOI:** 10.3389/fphar.2026.1784153

**Published:** 2026-03-18

**Authors:** Menghan Ye, Rui Zhang, Pengpeng Guo, Jinping Zhou, Dianwen Yu, Tianze Shang, Peixia Li, Jiaxin Li, Kaiyu Liu, Yani Liu, Shaojun Shi

**Affiliations:** Wuhan Union Hospital, Tongji Medical College, Huazhong University of Science and Technology, Wuhan, China

**Keywords:** fatty acid oxidation, IDO1, macrophage polarization, metabolic reprogramming, tacrolimus

## Abstract

Macrophage metabolic reprogramming toward the M1 phenotype is a key pathological feature of kidney injury. Recent studies have increasingly highlighted the importance of *de novo* NAD^+^ synthesis in the development of renal damage. In this study, we found that tacrolimus (TAC) suppressed the activity of indoleamine-2,3-dioxygenase 1 (IDO1), thereby blocking the conversion of tryptophan (Trp) to kynurenine (KYN), impairing *de novo* NAD^+^ synthesis. NAD^+^ deficiency enhances glycolysis, causes accumulation of medium-to long-chain fatty acids and acylcarnitines, indicating impaired fatty acid β-oxidation, thereby promoting M1 polarization and exacerbating renal injury. Further investigations revealed that restoring NAD^+^ levels via exogenous KYN supplementation or directly activating peroxisome proliferator-activated receptor alpha (PPARα) to enhance fatty acid oxidation effectively reversed this metabolic imbalance and alleviated TAC-induced kidney injury.

## Graphical Abstract

**Figure:**
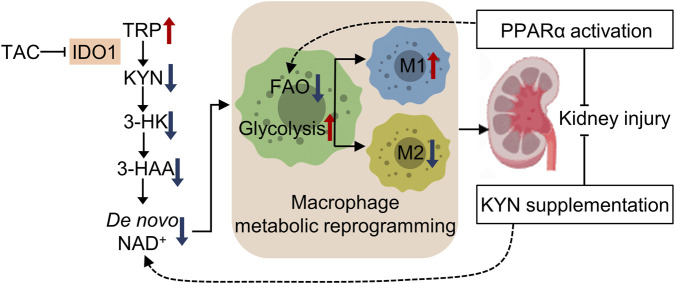

## 1 Introduction

Tacrolimus (TAC), a calcineurin inhibitor, is widely used to maintain immune tolerance after organ transplantation and has significantly improved post-transplant survival rates. However, TAC frequently leads to chronic kidney disease (CKD), characterized by progressive renal dysfunction, interstitial fibrosis, tubular atrophy, inflammatory cell infiltration, and apoptosis (Cao et al., 2023; Lim et al., 2019a; Ding et al., 2022). Previous studies on TAC-induced nephrotoxicity have mainly focused on oxidative stress, autophagy, and apoptosis. In recent years, increasing attention has been given to the interplay between metabolism and immune regulation in the context of CKD (Wang et al., 2021; Wang et al., 2025). Emerging evidence suggests that TAC not only promotes renal macrophage infiltration and polarization but also induces nephrotoxicity closely associated with NAD^+^ depletion (Shigematsu et al., 2022; Nishida et al., 2025). Macrophage metabolic reprogramming often precedes phenotypic changes and determines their functional fate (O’Neill and Pearce, 2016; Zeng et al., 2023). Proinflammatory M1 macrophages primarily rely on glycolysis for rapid ATP generation (Liu et al., 2021). Glycolysis enhances cytokine secretion and antimicrobial activity in M1 macrophages (Cervenka et al., 2017; Zhai et al., 2024), whereas anti-inflammatory M2 macrophages depend mainly on oxidative phosphorylation (OXPHOS) and fatty acid oxidation (FAO) to maintain their function.

Indoleamine-2,3-dioxygenase 1 (IDO1), an enzyme expressed in both renal tissue and macrophages, is predominantly distributed in extrahepatic tissues. IDO1 catalyzes the conversion of tryptophan (Trp) into kynurenine (KYN) and serves as the rate-limiting enzyme in the kynurenine pathway (KP). Trp is an essential amino acid that cannot be synthesized by the body and is primarily degraded by tryptophan-2,3-dioxygenase (TDO2) in the liver or by IDO1 and the recently identified IDO2 in peripheral tissues (Figure 3G) (Cervenka et al., 2017). IDO1 activity is markedly upregulated in response to various immunogenic stimuli, including lipopolysaccharide (LPS) and proinflammatory cytokines. Recent studies have shown that IDO1 helps sustain the high energy demands of human embryonic stem cells (hESCs) and hippocampal neurons by enhancing glycolysis or lactate production (Zhai et al., 2024; Wang et al., 2023). The expression of IDO1 has also been reported to influence macrophage differentiation (Ji et al., 2025; Liu et al., 2025). However, the role of IDO1 in regulating macrophage metabolism has not been thoroughly investigated.

KYN is a key metabolic intermediate with immunomodulatory, antioxidant, and neuroactive properties. As a critical product of the KP, KYN can be further metabolized to quinolinic acid (QA), which serves as a precursor for *de novo* NAD^+^ synthesis. *De novo* NAD^+^—generated from the Trp-derived pathway—plays a central role in both glycolysis and oxidative phosphorylation by transferring electrons to drive ATP production. Disruption of NAD^+^ homeostasis in macrophages impairs mitochondrial function, promotes glycolysis, and facilitates M1 polarization and inflammatory responses. Although the KP is one of the major sources of *de novo* NAD^+^, its role in NAD^+^ generation has been underappreciated due to a predominant focus on Trp metabolites and neuroactive intermediates (Wee et al., 2021). To date, *de novo* NAD^+^ synthesis has been considered mainly a hepatic process, with peripheral tissues relying on liver-derived nicotinamide to meet their NAD^+^ demands. Recent studies demonstrate that KP-dependent *de novo* NAD^+^ synthesis is critical for renal and macrophage metabolism, and its enhancement improves mitochondrial function and alleviates kidney injury *in vitro* (Wang et al., 2022; Poyan et al., 2018; Minhas et al., 2019). Although the contribution of *de novo* NAD^+^ in peripheral tissues is generally low, it accounts for over 40% of total NAD^+^ in macrophages, supporting OXPHOS, FAO, and the activity of Sirtuins (Minhas et al., 2019).

Based on these findings, the present study aimed to demonstrate, through both *in vivo* and *in vitro* models, that TAC targets IDO1 to impair *de novo* NAD^+^ synthesis, thereby driving metabolic reprogramming of macrophage energy metabolism at both the transcriptomic and metabolic levels.

## 2 Methods

### 2.1 Mice

Eight-week-old male C57BL/6J mice were obtained from Beijing Vital River Laboratory Animal Technology Co., Ltd. Hubei Branch (Wuhan, China) under license number SCXK (E) 2022–0030. All animals were acclimated for 1 week prior to experimentation. Mice were housed in a specific pathogen-free (SPF) facility at the Laboratory Animal Center of Huazhong University of Science and Technology. Environmental conditions were maintained at a temperature of 20 °C–22 °C with a relative humidity of 40%–60% and a 12-h light/dark cycle. Food and water were provided *ad libitum* throughout the acclimation and experimental periods. All animal procedures followed international ethical guidelines and were approved by the Animal Ethics Committee of Huazhong University of Science and Technology (IACUC No. 5061, 2025).

### 2.2 Animals study design

Male C57BL/6J mice (8 weeks old) were randomly assigned to five experimental groups: control (CON), TAC (MedChemExpress, Cat# HY-13756), TAC + KYN (Sigma-Aldrich, Cat# K8625), 1-methyl-D-tryptophan (1-MT, MedChemExpress, Cat# HY-107946), and TAC + pemafibrate (Selleck, Cat# S8655). Mice received treatments for 8 weeks. TAC was administered at 2 mg/kg/day by intraperitoneal (i.p.) injection. This dosage was selected based on established chronic tacrolimus nephrotoxicity models in rodents (Lim et al., 2019a; Yu et al., 2019; Luo et al., 2019). KYN was administered at 200 mg/kg every other day (i.p.); 1-MT at 10 mg/kg/day (i.p.); and pemafibrate at 0.5 mg/kg/day by oral gavage (p.o.). CON mice received the corresponding vehicles matched for route and dosing schedule.

At the end of the 8-week treatment period, all mice were fasted for 12 h with free access to water. Urine was collected from individually housed mice over 24 h in individual metabolic cages prior to euthanasia, transferred to prechilled tubes, centrifuged at 3,000 rpm for 10 min to remove debris, aliquoted, and stored at −80 °C until analysis. Mice were then euthanized, and blood was harvested by cardiac puncture into anticoagulant-coated tubes. Plasma was obtained by centrifugation at 3,000 rpm for 15 min. Kidneys were isolated, rinsed with phosphate-buffered saline (PBS, pH 7.4), and either snap-frozen in liquid nitrogen for biochemical and molecular assays or fixed in 4% paraformaldehyde for histological analysis.

### 2.3 Renal function and histopathological assessment

Renal function was assessed by measuring creatinine (C011-2-1), urea nitrogen (C013-2-1), uric acid (C012-1-1) and urinary protein (C035-2-1) using commercial kits from Nanjing Jiancheng Bioengineering Institute (Nanjing, China), following the manufacturer’s protocols.

Renal cortex tissues were fixed in 4% paraformaldehyde, embedded in paraffin, and cut into 4 μm sections. Standard hematoxylin and eosin (HE) and periodic acid–Schiff (PAS) staining were performed to evaluate general renal morphology and tubular injury. All slides were examined under a light microscope, and representative pathological changes were assessed qualitatively in a blinded manner. Tubular injury was quantified using a semi-quantitative scoring system based on tubular epithelial cell degeneration and necrosis, tubular dilation, intraluminal cast formation, and inflammatory cell infiltration, with scores ranging from 0 to 4 (0 = normal; 1 = <25%; 2 = 25–50%; 3 = 50–75%; 4 = >75% of tubules showing injury) (Weidemann et al., 2008).

### 2.4 Cell culture

The immortalized murine bone marrow–derived macrophage cell line (iBMDM) was obtained from Cyagen Biosciences Inc. (OriCell®, Guangzhou, China; Cat. No. M3-1001). The human embryonic kidney 293T (HEK293T) cell line was obtained from Wuhan Pricella Biotechnology Co., Ltd. (Procell, Wuhan, China; Cat. No. CL-0005). Both cell lines were cultured in high-glucose Dulbecco’s Modified Eagle Medium (DMEM-H; Gibco) supplemented with 10% fetal bovine serum (FBS; Gibco) and 1% penicillin–streptomycin (P/S; Gibco), and maintained at 37 °C in a humidified 5% CO_2_ incubator. For *in vitro* treatments, cells were exposed to TAC (15 μM), 1-MT (200 μM), KYN (100 μM), or pemafibrate (40 μM) for 24 h as indicated. CON cells received a vehicle cocktail matching the solvent composition and volume of the corresponding treatment conditions (e.g., DMSO for TAC/pemafibrate stocks and PBS for KYN/1-MT), with a final DMSO concentration ≤0.1% (v/v).

### 2.5 Immunofluorescence staining

Paraffin-embedded renal cortex tissues were sectioned at 5 μm thickness, deparaffinized in xylene, and rehydrated through a graded ethanol series. Antigen retrieval was performed using citrate buffer (pH 6.0) at 95 °C for 20 min. After cooling to room temperature, sections were blocked with 3% BSA in PBS for 30 min. Double immunofluorescence staining was conducted by incubating sections overnight at 4 °C with primary antibody pairs: rabbit anti-CD68 (1:300) combined with mouse anti-CD86 (1:200) to identify M1 macrophages, or rabbit anti-CD68 (1:300) with goat anti-ARG1 (1:200) to label M2 macrophages (all antibodies from Servicebio, China). After washing with PBS, appropriate species-specific secondary antibodies conjugated to Alexa Fluor 488 or 594 were applied for 1 h at room temperature. Nuclei were counterstained with DAPI. Images were captured using a Leica fluorescence microscope, and the co-localization of CD68 with CD86 or ARG1 was quantified using ImageJ software (version 1.8.0).

### 2.6 RT-qPCR

Total RNA was extracted from cells using TRIzol reagent (Invitrogen, Carlsbad, CA, United States). cDNA was synthesized using the Hifair® III 1st Strand cDNA Synthesis SuperMix (YEASEN, Shanghai, China; Cat# 11137ES10) according to the manufacturer’s instructions. RT-qPCR was performed using SYBR Green Master Mix on a real-time PCR system (Bioer, Hangzhou, China). β-Actin was used as the internal control. Primer sequences are listed in Supplementary Table S1.

### 2.7 Western blot

Renal cortical tissues were lysed in RIPA buffer supplemented with protease inhibitor cocktail, phosphatase inhibitor cocktail, and phenylmethylsulfonyl fluoride (PMSF). Total protein concentrations were determined using a BCA assay kit (Beyotime Biotechnology). Protein samples were boiled at 95 °C for 10 min, separated on 12% SDS–PAGE gels, and then transferred onto polyvinylidene fluoride (PVDF) membranes. Membranes were blocked with 5% nonfat milk in TBST at room temperature for 1 h and subsequently incubated with primary antibodies at 4 °C overnight. After washing with TBST three times (5 min each), membranes were incubated with horseradish peroxidase (HRP)–conjugated secondary antibodies for 2 h at room temperature. Protein bands were visualized using enhanced chemiluminescence. Primary antibodies used in this study included: IDO1 (Proteintech, Cat# 13268-1-AP, 1:1000), CPT1 (ABclonal, Cat# A5307, 1:2000), CPT2 (Proteintech, Cat# 26555-1-AP, 1:8000), CACT (ABclonal, Cat# A13956, 1:400), β-tubulin (Proteintech, Cat# 80713-1-RR, 1:10,000), HRP-conjugated Goat Anti-Rabbit IgG (H + L) (ABclonal, Cat# SA00001-2, 1:10,000).

### 2.8 Untargeted metabolomics

Untargeted metabolomic profiling was performed using an ultra-performance liquid chromatography coupled with quadrupole time-of-flight mass spectrometry system (UPLC-Q-TOF, Waters, Shanghai, China). Chromatographic separation was achieved on an ACQUITY UPLC HSS T3 column (100 Å, 1.8 μm, 1.0 × 100 mm, Waters). The mobile phases consisted of 0.1% formic acid in water (A) and 0.1% formic acid in acetonitrile (B). The flow rate was set at 0.3 mL/min, with the column maintained at 40 °C and the autosampler at 4 °C. The injection volume was 5 μL. The gradient elution program was as follows: 0 min, 5% B; 1 min, 5% B; 11 min, 100% B; 13 min, 100% B; 15 min, 5% B; 20 min, 5% B, for a total run time of 20 min. Mass spectrometry was performed in both positive and negative ion modes. The capillary voltage was set at 3.0 kV for positive mode and 2.5 kV for negative mode. Other parameters were as follows: source temperature, 300 °C; desolvation temperature, 400 °C; cone gas flow rate, 800 L/h; sample cone voltage, 40 V; and source offset voltage, 80 V. Mass spectra were acquired over an m/z range of 50–1200 Da with a scan time of 1 s in continuum mode. Data acquisition and instrument control were performed using MassLynx software. For plasma sample preparation, 100 μL of plasma was mixed with 400 μL of methanol/acetonitrile (60:40, v/v), vortexed for 30 min, and centrifuged at 14,000 rpm for 10 min at 4 °C. The supernatant was filtered through a 0.45 μm membrane and subjected to LC-MS analysis. For renal cortex samples, approximately 50 mg of renal cortex tissue was homogenized in 300 μL of methanol/acetonitrile (60:40, v/v), vortexed, centrifuged, and filtered prior to analysis.

### 2.9 Fatty acid metabolomics analysis

Fatty acid profiles in renal cortex tissue and cellular pellets were quantified using ultra-performance liquid chromatography coupled with tandem mass spectrometry (UPLC-MS/MS, QTRAP 5500, AB Sciex). Chromatographic separation was performed on an ACQUITY BEH C18 column (100 mm × 2.1 mm, 1.7 μm; Waters) at a flow rate of 0.3 mL/min. Briefly, 100 mg of renal cortex tissue or 10^7^ cells were homogenized in 1 mL of ice-cold extraction solvent (methanol: dichloromethane: n-hexane = 1:1:1, v/v/v) containing the internal standard Stearic Acid-d35. After centrifugation, the supernatant was collected and vacuum-dried for 2 h. The residue was reconstituted in 60 μL methanol, followed by derivatization with 30 μL of 3-nitrophenylhydrazine (200 mM in 70% methanol) and 30 μL of EDC (120 mM in 70% methanol) at 40 °C for 60 min. The reaction mixtures were centrifuged and the supernatant was injected into the UPLC-MS system. The mobile phases consisted of 0.1% formic acid in water (solvent A) and acetonitrile/isopropanol (7:3, v/v, solvent B). The gradient program was as follows: 0–1 min, 50% B; 1–3.5 min, linear increase to 78% B; 3.5–12.2 min, linear increase to 100% B; 12.2–14.2 min, 100% B; 14.2–14.5 min, linear decrease to 50% B; 14.5–16 min, 50% B. Mass spectrometry was performed in negative ionization mode with a capillary voltage of −2.0 kV, source temperature of 150 °C, and desolvation temperature of 550 °C.

### 2.10 Energy metabolomics analysis

Targeted profiling of energy metabolism intermediates in renal cortex tissue and cellular pellets was performed using ultra-performance liquid chromatography-tandem mass spectrometry (UPLC-MS/MS, QTRAP 5500, AB Sciex). Chromatographic separation was conducted on an ACQUITY BEH Amide column (100 mm × 2.1 mm, 1.7 μm; Waters) with a flow rate of 0.3 mL/min. Briefly, 100 mg of renal cortex tissue or 10^7^ cells were homogenized in 1 mL of ice-cold extraction solvent (methanol: acetonitrile: water = 2:2:1, v/v/v) containing succinic acid-d4 as the internal standard. After homogenization and vortexing, samples were centrifuged, and the supernatant was vacuum-dried for 2 h. The dried extracts were reconstituted in methanol: acetonitrile (1:1, v/v) and subjected to UPLC-MS/MS analysis. The mobile phase A was 0.1% formic acid and 30 mM ammonium formate in water, and mobile phase B was 0.1% formic acid in acetonitrile. The elution gradient was programmed as follows: 0–1 min, 5% B; 1–11 min, linear increase to 100% B; 11–13 min, 100% B; 13–15 min, linear decrease to 5% B; 15–20 min, 5% B. Mass spectrometry was performed in negative ion mode.

### 2.11 Trp metabolomics analysis

Trp metabolites in plasma, renal cortex tissue, and cellular pellets were quantified using ultra-performance liquid chromatography-tandem mass spectrometry (UPLC-MS/MS, QTRAP 5500, AB Sciex). Chromatographic separation was performed on an ACQUITY UPLC HSS T3 column (100 mm × 1.0 mm, 1.8 μm, 100 Å; Waters) maintained at 40 °C with a flow rate of 0.3 mL/min. For renal cortex tissue and cell pellets, 100 mg of tissue or 1 × 10^7^ cells were homogenized in 300 μL of 6% methanolic hypochlorous acid, followed by addition of 300 μL of methanol containing internal standards. Samples were vortexed and centrifuged, and the supernatant was collected for analysis. For plasma samples, 100 μL of plasma was mixed with 600 μL of 6% methanolic hypochlorous acid containing internal standards, vortexed, and centrifuged to obtain the supernatant. The mobile phase A consisted of 0.1% formic acid in water, and mobile phase B was acetonitrile. The elution gradient was set as follows: 0–1 min, 5% B; 1–11 min, linear increase to 100% B; 11–13 min, 100% B; 13–15 min, linear decrease to 5% B; 15–20 min, 5% B. Mass spectrometry was operated in positive ion mode.

### 2.12 Isolation of renal macrophages

Renal macrophages were isolated from mouse kidneys as previously described (Jia et al., 2025). Briefly, dissected kidneys were minced and enzymatically digested with collagenase/DNase to prepare a single-cell suspension. After red blood cell lysis and washing, leukocytes were enriched and renal macrophages were isolated by magnetic-activated cell sorting using a Mouse F4/80 Positive Selection Kit (Stemcell Technologies) according to the manufacturer’s instructions. Enriched F4/80^+^ macrophages were used for downstream assays, including RNA extraction and qPCR.

### 2.13 Mitochondrial bioenergetics and metabolic assays

Mitochondrial respiration was assessed using the Seahorse XF24 Extracellular Flux Analyzer (Agilent Technologies, Loveland, CO, United States). To evaluate both exogenous and endogenous FAO, BSA-conjugated palmitate was prepared following the manufacturer’s protocol (Agilent Seahorse Biosciences). For experiments utilizing glucose as the primary carbon source, the culture medium was replaced with one containing 25 mM glucose. For palmitate-driven respiration, the medium was modified to include 0.5 mM glucose and 0.23 mM BSA-conjugated palmitate. Sequential injections of oligomycin (1 μM), carbonyl cyanide-p-trifluoromethoxyphenylhydrazone (FCCP; 2 μM), and a mixture of rotenone and antimycin A (1 μM each) were performed to assess mitochondrial function. All oxygen consumption rate (OCR) values were normalized to total protein content. Substrate-specific assays: glucose vs. palmitate.

### 2.14 Molecular docking analysis

The crystal structure of human IDO1 (PDB ID: 4U72) was obtained from the Protein Data Bank (PDB). The chemical structure of tacrolimus was retrieved from PubChem. Molecular docking analysis was performed using the CB-Dock online server (http://clab.labshare.cn/cb-dock/php/index.php). The binding interactions between tacrolimus and IDO1 were further visualized using PyMOL software and the Proteins Plus online server (https://proteins.plus).

### 2.15 Statistical analysis

Statistical analyses were conducted using GraphPad Prism 8 (GraphPad Software, La Jolla, CA, United States). Data are presented as mean ± SEM, with a minimum of five independent replicates per group. For targeted metabolomics data, metabolite concentrations from renal cortex tissue were normalized to the wet tissue weight (pmol/mg tissue) prior to statistical analysis. Comparisons among multiple groups were performed using one-way analysis of variance (ANOVA) followed by Tukey’s post hoc test. For comparisons between two groups, statistical significance was determined using two-tailed Student’s t-test with unequal variance. A P-value less than 0.05 was considered statistically significant.

## 3 Result

### 3.1 TAC disrupts Trp metabolism in the kidney

**FIGURE 1:**
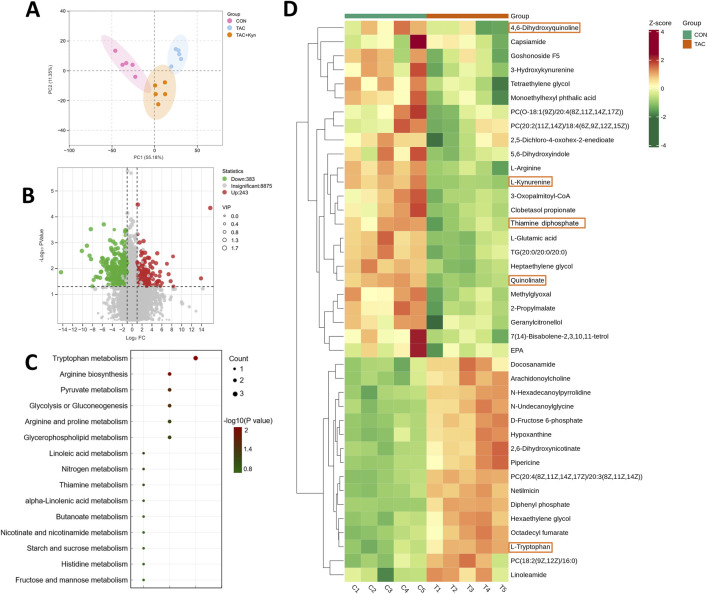
Metabolomic alterations in the kidney following TAC treatment **(A)** Principal component analysis (PCA) showing the distribution of metabolic profiles among the CON, TAC, and TAC + KYN groups. **(B)** Volcano plot illustrating differential metabolites between the TAC and CON groups (VIP > 1, P < 0.05). **(C)** KEGG pathway enrichment analysis based on the top 50 most abundant differential metabolites with P < 0.05. **(D)** Heatmap of the top 50 most abundant differential metabolites with P < 0.05.

To investigate the effects of TAC on renal metabolic networks, we performed untargeted metabolomic profiling of renal cortex from control (CON), TAC-treated, and TAC + KYN-treated mice. Principal component analysis (PCA) revealed a clear separation among the three groups, indicating that TAC induces substantial metabolic remodeling in renal cortex, while KYN supplementation partially restored the metabolic profile toward that of the CON group (Figure 1A).

Volcano plot analysis comparing the CON and TAC groups identified numerous metabolites that were significantly altered following TAC treatment (Figure 1B). Kyoto Encyclopedia of Genes and Genomes (KEGG) pathway enrichment analysis showed that 15 metabolic pathways were significantly affected (P < 0.05), including Trp metabolism, glycolysis, and pyruvate metabolism. Among them, Trp metabolism was the most significantly disturbed (Figure 1C). To further visualize intergroup metabolic changes, we generated a hierarchical clustering heatmap based on the top 60 differentially abundant metabolites between the CON and TAC groups. Notably, metabolites involved in the Trp–KP exhibited a distinct shift in TAC-treated mice. Trp levels were increased, whereas downstream metabolites including KYN and quinolinate were markedly decreased, indicating impaired flux through the Trp–KYN pathway and potential suppression of *de novo* NAD^+^ synthesis (Figure 1D). In addition, alterations in energy-related metabolites, such as thiamin diphosphate and D-fructose 6-phosphate, further suggested disruption of glycolysis and mitochondrial metabolic homeostasis. These coordinated metabolic changes indicate that TAC-induced nephrotoxicity is associated with dysregulation of Trp metabolism and renal energy balance.

### 3.2 TAC specifically inhibits Ido1 expression to induce kidney injury

**FIGURE 2:**
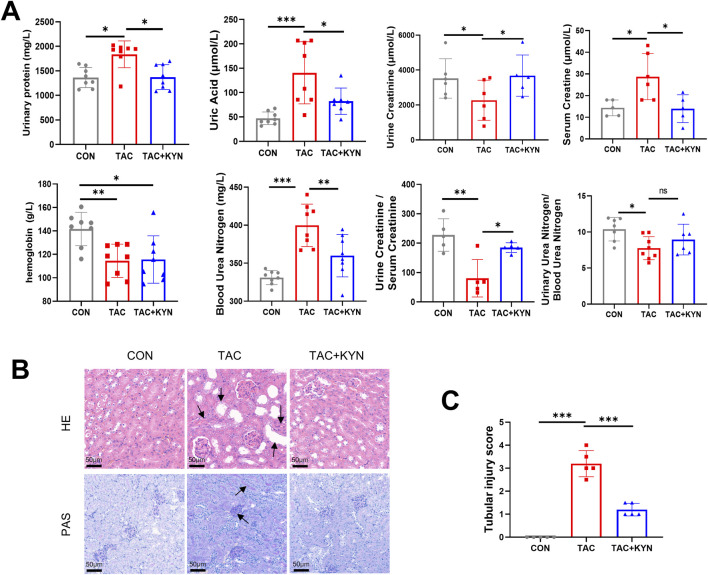
KYN reverses TAC-induced kidney injury **(A)** Biochemical parameters of renal function in mice, including urinary protein, uric acid, serum creatinine, urinary creatinine, hemoglobin, blood urea nitrogen, urine creatinine/serum creatinine ratio, and urinary urea nitrogen/blood urea nitrogen ratio (n = 8). **(B)** Representative H&E, Masson, and PAS staining images of kidney sections from mice. Arrows highlight tubular injury features. Scale bar = 50 μm. **(C)** Quantification of tubular injury scores based on histological assessment (n = 5). Data are presented as mean ± SD. Statistical analyses were performed using one-way ANOVA followed by Tukey’s multiple comparisons test. *P < 0.05, **P < 0.01, ***P < 0.001.

To explore the potential protective effect of correcting Trp metabolic disturbances in TAC-induced kidney injury, three groups of mice (CON, TAC, and TAC + KYN) were treated for eight consecutive weeks, and renal function was evaluated. Compared with the CON group, TAC significantly reduced urinary creatinine levels and increased urinary protein, serum creatinine, blood urea nitrogen, and uric acid levels. These alterations were significantly reversed by KYN co-administration (Figure 2A). Histological analysis using H&E and PAS staining revealed that TAC induced marked tubular injury, including tubular epithelial degeneration, tubular dilation, intraluminal protein cast formation, and inflammatory cell infiltration (Figure 2B). KYN treatment attenuated these morphological abnormalities and decreased tubular injury scores (Figures 2B,C).

**FIGURE 3:**
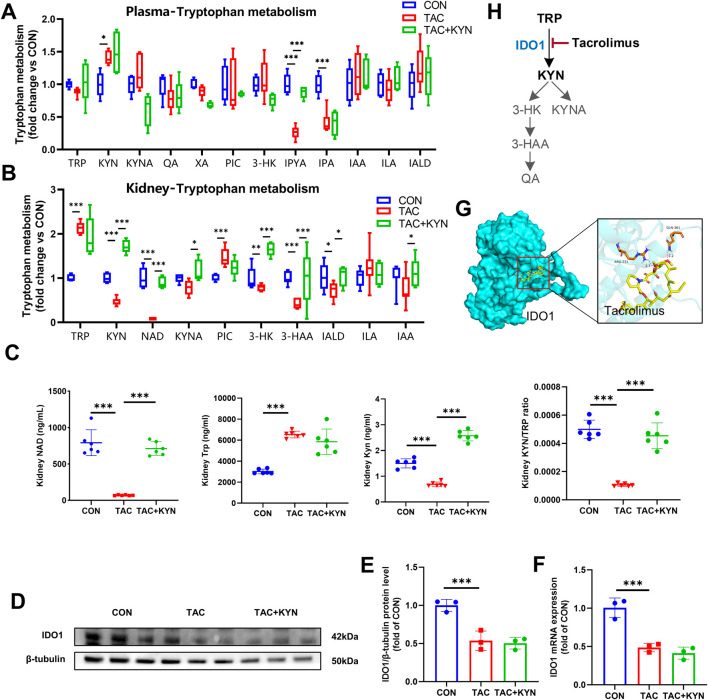
TAC Specifically Inhibits IDO1 Expression to Induce Kidney Injury **(A)** Relative abundances of Trp metabolites in mouse plasma (n = 5). **(B)** Relative abundances of Trp metabolites in the renal cortex (n = 5). **(C)** Quantification of Trp, KYN, the KYN/Trp ratio, and NAD^+^ levels in renal tissues (n = 5). **(D)** Western blot analysis of IDO1 protein expression in the renal cortex, with β-tubulin as a loading control. **(E)** Densitometric quantification of IDO1 protein bands (n = 3). **(F)** qPCR analysis of IDO1 mRNA expression in the renal cortex (n = 3). **(G)** Molecular docking model of TAC binding to IDO1. **(H)** Schematic illustration showing that IDO1 inhibition disrupts the Trp–KYN pathway, affecting downstream metabolites including 3-HK, KYNA, 3-HAA, and QA. Data are presented as mean ± SD. Statistical analyses were performed using one-way ANOVA followed by Tukey’s multiple comparisons test. *P < 0.05, **P < 0.01, ***P < 0.001. Abbreviations: TRP, tryptophan; KYN, kynurenine; KYNA, kynurenic acid; QA, quinolinic acid; XA, xanthurenic acid; PIC, picolinic acid; 3-HK, 3-hydroxykynurenine; 3-HAA, 3-hydroxyanthranilic acid; IPYA, indole-3-pyruvic acid; IPA, indole-3-propionic acid; ILA, indolelactic acid; IALD, indoleacetaldehyde; IAA, indoleacetic acid; NAD^+^, nicotinamide adenine dinucleotide.

To clarify the alterations in Trp metabolism induced by TAC, targeted metabolomic profiling was performed across the three groups (CON, TAC, and TAC + KYN). In plasma, TAC markedly increased KYN, whereas the key intermediates of *de novo* NAD^+^ synthesis, including 3-hydroxykynurenine (3-HK) and quinolinic acid (QA), remained unchanged. Other metabolites from different branches of the KP, such as kynurenic acid (KYNA), xanthurenic acid (XA), and picolinic acid (PIC), also showed no significant variation (Figure 3A). In contrast, indole-branch metabolites indole-3-pyruvic acid (IPYA) and indole propionic acid (IPA) were significantly reduced and partially restored by KYN supplementation.

In the renal cortex, TAC produced a characteristic pattern of suppression of *de novo* NAD^+^ synthesis. TAC markedly increased Trp levels (+119%) while significantly reducing several key metabolites along the KP, including KYN (−54.2%), 3-HK, 3-hydroxyanthranilic acid (3-HAA), as well as the final product NAD^+^ (−91.7%) (Figure 3B). The KYN/Trp ratio, a classical surrogate marker of indoleamine 2,3-dioxygenase 1 (IDO1) activity, was also markedly decreased (Figure 3C), indicating substantial inhibition of IDO1. Meanwhile, elevated PIC suggested branch diversion within the KP, whereas reductions in indoleacetaldehyde (IALD) and indolelactic acid (ILA), along with a mild increase in indoleacetic acid (IAA), reflected dysregulation of downstream metabolic branches. Following KYN supplementation, KYN, 3-HK, 3-HAA, and NAD^+^ were all significantly restored, and the KYN/Trp ratio returned toward normal levels, indicating that TAC-induced inhibition of IDO1 and the downstream IDO1-dependent *de novo* NAD^+^ synthesis was effectively reversed.

RT-qPCR and Western blot analysis further confirmed that TAC significantly suppressed renal IDO1 expression (Figures 3D–F). Molecular docking analysis also demonstrated that TAC can specifically target IDO1 (Figure 3G). Collectively, these findings indicate that TAC is associated with downregulation of IDO1 expression in the renal cortex, thereby contributing to suppression of *de novo* NAD^+^ synthesis (Figure 3H).

### 3.3 IDO1 inhibition disrupts fatty acid oxidation and impairs renal energy metabolism

**FIGURE 4:**
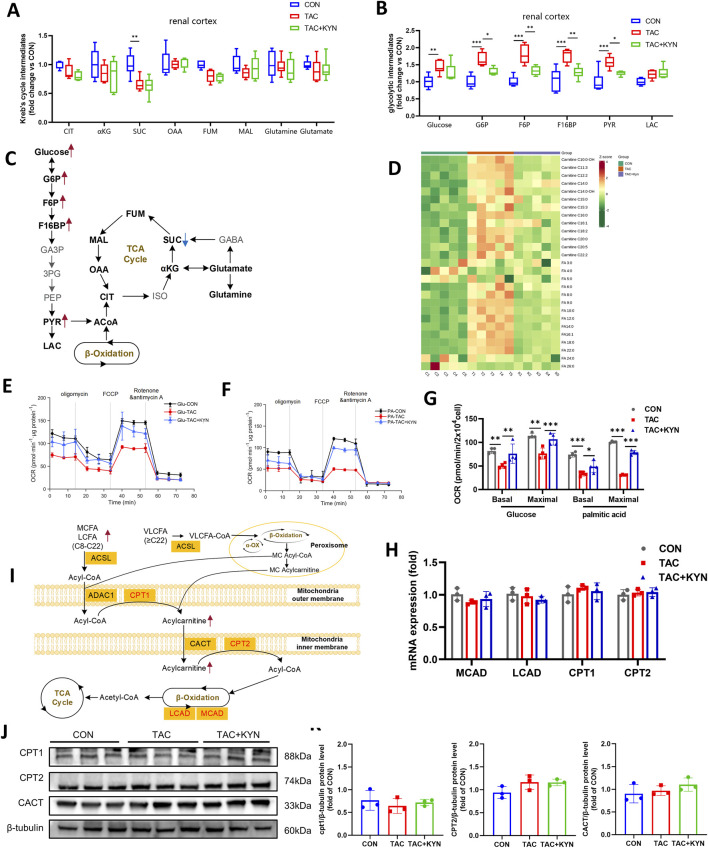
KYN supplementation alleviates TAC-induced energy metabolism disorder by restoring FAO. **(A)** Relative abundance of TCA cycle–related metabolites in the renal cortex (n = 5). **(B)** Relative abundance of glycolysis-related metabolites in the renal cortex (n = 5). **(C)** Schematic diagram of energy metabolism pathways based on metabolomic data, including the TCA cycle, glycolysis, and β-oxidation; red arrows indicate metabolites upregulated by TAC treatment, and blue arrows indicate metabolites downregulated by TAC treatment. **(D)** Heatmap of fatty acid metabolism–related compounds in the renal cortex (n = 5). **(E,F)** Seahorse analysis showing mitochondrial oxygen consumption rate (OCR) in renal cortical cells using glucose **(E)** or palmitic acid **(F)** as substrates (n = 4). **(G)** Quantification of basal and maximal OCR in renal cortical cells under glucose or palmitic acid substrate conditions (n = 4). **(H)** RT-qPCR analysis of FAO-related gene expression (MCAD, LCAD, CPT1, CPT2) in the renal cortex (n = 3). **(I)** Schematic diagram of mitochondrial fatty acid transport and β-oxidation pathways. **(J)** Western blot analysis of CPT1, CPT2, and CACT protein expression in the renal cortex, with β-tubulin as a loading control. **(K)** Densitometric quantification of CPT1, CPT2, and CACT protein bands from Western blots (n = 3). Data are presented as mean ± SD. Statistical significance is indicated as *P < 0.05, **P < 0.01, ***P < 0.001. Abbreviations: CIT, citrate; αKG, alpha-ketoglutarate; SUC, succinate; OAA, oxaloacetate; FUM, fumarate; MAL, malate; PYR, pyruvate; LAC, lactate; G6P, glucose-6-phosphate; F6P, fructose-6-phosphate; F1,6BP, fructose-1,6-bisphosphate; MCAD, medium-chain acyl-CoA dehydrogenase; LCAD, long-chain acyl-CoA dehydrogenase; CPT1/2, carnitine palmitoyltransferase 1/2; CACT, carnitine-acylcarnitine translocase; ACSL, acyl-CoA synthetase long-chain family member; OCR, oxygen consumption rate.

Given the critical role of IDO1 and its associated *de novo* NAD^+^ synthesis in maintaining cellular energy homeostasis (Xie et al., 2020), and in light of the substantial alterations in energy metabolites revealed by untargeted metabolomics, we further performed targeted energy metabolomic profiling of the renal cortex. IDO1 inhibition significantly increased the levels of glucose, glucose-6-phosphate (G6P), fructose-6-phosphate (F6P), fructose-1,6-bisphosphate (F1,6BP), and pyruvate (PYR), indicating enhanced aerobic glycolysis, whereas KYN supplementation effectively reversed the accumulation of these intermediates. In the tricarboxylic acid (TCA) cycle, TAC treatment resulted in a decrease in succinate (SUC), and this reduction was not corrected by KYN (Figures 4A–C). Although TAC reduced SUC levels, the lack of recovery following KYN treatment suggests that this selective alteration in the TCA cycle is unlikely to be primarily mediated by IDO1-dependent *de novo* NAD^+^ synthesis. Notably, other TCA-related metabolites such as citrate, α-ketoglutarate, malate, and the anaplerotic substrates glutamate and glutamine remained largely unchanged, supporting that overall TCA cycle activity was preserved.

Regarding FAO, TAC treatment caused marked accumulation of medium- and long-chain acylcarnitines (C10–C22) and medium-to long-chain free fatty acids (C8–C22), while short-chain (C3–C6) and very long-chain fatty acids (C24–C25) remained unchanged. Because medium- and long-chain fatty acids undergo mitochondrial β-oxidation, their selective accumulation indicates that TAC predominantly impairs mitochondrial β-oxidation. KYN supplementation effectively reduced the accumulation of these fatty acids and acylcarnitines, further supporting that TAC-induced β-oxidation defects arise from IDO1 inhibition and the resulting insufficiency of *de novo* NAD^+^ supply (Figure 4D).

We next isolated mouse renal cortical cells and measured OCR using either glucose or palmitate as the primary substrate. TAC significantly suppressed oxidative phosphorylation under both substrate conditions, as evidenced by reduced basal and maximal respiration, whereas exogenous KYN restored these defects (Figures 4E,F). Notably, the decline in maximal respiration was more pronounced when palmitate served as the main substrate, indicating that TAC preferentially impairs FAO and thereby disrupts mitochondrial energy metabolism (Figure 4G).

To determine whether impaired β-oxidation resulted from transcriptional regulation of FAO-related enzymes, we measured expression of medium-chain acyl-CoA dehydrogenase (MCAD), long-chain acyl-CoA dehydrogenase (LCAD), and carnitine palmitoyltransferases 1 and 2 (CPT1, CPT2). No significant differences were observed among groups (Figures 4H,I). Western blot analysis further confirmed that the protein levels of CPT1, CPT2, and carnitine-acylcarnitine translocase (CACT) were unchanged following KYN administration (Figures 4J,K). These findings suggest that the KYN-mediated improvement in fatty acid β-oxidation is unlikely to rely on transcriptional or translational regulation of FAO enzymes, but rather reflects restoration of overall metabolic homeostasis.

### 3.4 Ido1 inhibition induces kidney injury by driving macrophage metabolic reprogramming

**FIGURE 5:**
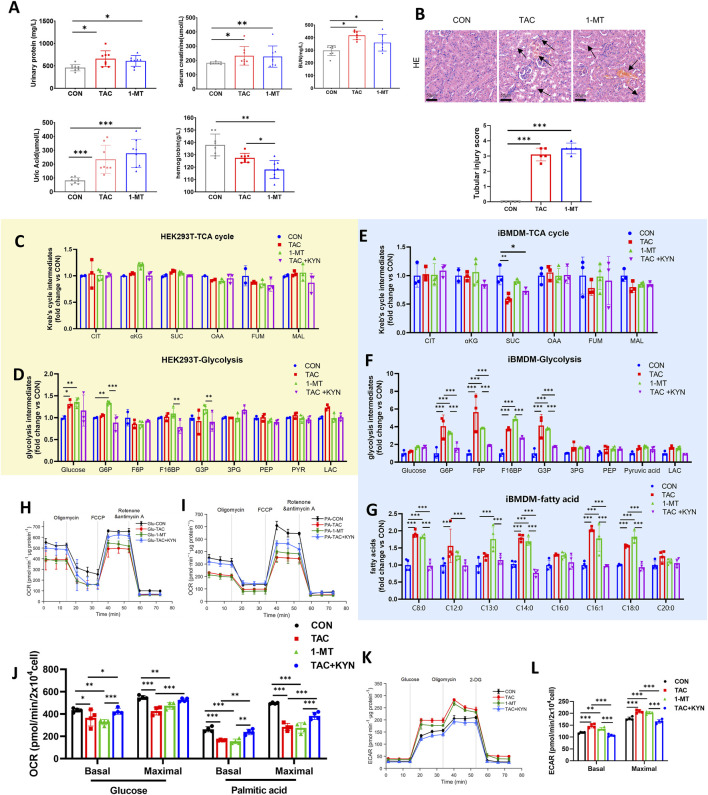
Indicators of renal function and energy metabolism upon IDO1 inhibition. **(A)** Biochemical indicators of kidney injury in mice, including urinary protein, serum creatinine, blood urea nitrogen, uric acid, and hemoglobin (n = 8). **(B)** Representative H&E staining of kidney sections and corresponding tubular injury scores (n = 5). Arrows highlight tubular injury features. Scale bar = 50 μm. **(C,D)** Targeted analysis of TCA cycle metabolites **(C)** and glycolytic metabolites **(D)** in HEK293T cells. **(E,F)** Targeted analysis of TCA cycle metabolites **(E)** and glycolytic metabolites **(F)** in iBMDMs. **(G)** Targeted measurement of fatty acid–related metabolites in iBMDMs. **(H)** OCR trace of iBMDMs measured using glucose as the substrate during a Seahorse mitochondrial stress test (n = 6). **(I)** OCR trace of iBMDMs measured using palmitic acid as the substrate during a Seahorse mitochondrial stress test (n = 6). **(J)** Quantification of basal and maximal OCR in iBMDMs using glucose or palmitic acid as substrates. **(K)** ECAR trace of iBMDMs during glycolysis stress testing (n = 6). **(L)** Quantification of basal and maximal ECAR in iBMDMs. Data are presented as mean ± SD. Statistical analyses were performed using one-way ANOVA followed by Tukey’s multiple comparisons test. *P < 0.05, **P < 0.01, ***P < 0.001.

To further investigate the effect of IDO1 inhibition on renal injury, three groups of mice were established: CON, TAC, and a group treated with the specific IDO1 inhibitor 1-MT. Biochemical analyses showed that both TAC and 1-MT treatment resulted in significant renal dysfunction compared with the CON group (Figure 5A). Histological examination using H&E staining revealed marked tubular injury in both TAC- and 1-MT–treated mice (Figure 5B), indicating that renal damage induced by TAC is consistent with that caused by direct IDO1 inhibition.

Given the distinct roles of renal tubular epithelial cells and macrophages in kidney energy metabolism and inflammatory regulation, we next evaluated the metabolic effects of TAC and 1-MT in renal tubular epithelial cells (HEK293T) and macrophages (iBMDM) to determine cell-type specificity. In HEK293T cells, neither TAC nor 1-MT significantly altered intermediates of glycolysis or the TCA cycle, suggesting minimal impact of TAC on basal energy metabolism in tubular epithelial cells (Figures 5C,D).

In contrast, iBMDMs displayed enhanced glycolysis and reduced FAO. While overall TCA cycle activity remained largely unchanged, TAC treatment induced a selective decrease in SUC, which could not be restored by KYN, whereas 1-MT did not induce a similar reduction (Figures 5E,F). This divergence suggests that TAC-induced SUC reduction may be partially independent of IDO1 inhibition and could instead be related to additional immunosuppressive or mitochondrial toxic effects of TAC, indicating that SUC changes are not a primary feature of the IDO1-mediated Trp–KP and *de novo* NAD^+^ synthesis. Lipidomic analysis further showed prominent accumulation of medium-chain and long-chain free fatty acids (C8–C18) in iBMDMs, indicating impaired fatty acid β-oxidation (Figure 5G). KYN supplementation effectively reversed the accumulation of glycolytic intermediates and free fatty acids, thereby partially restoring β-oxidation capacity and alleviating the glycolytic shift.

We next performed mitochondrial respiration analysis in iBMDMs. IDO1 inhibition significantly reduced OCR, with a more pronounced decrease observed when palmitate was used as the primary substrate, indicating further impairment of fatty acid β-oxidation (Figures 5H–J). Meanwhile, ECAR levels were elevated, suggesting increased glycolytic activity; KYN supplementation partially corrected this metabolic imbalance (Figures 5K,L).

Taken together, these findings demonstrate that IDO1 inhibition induces energy metabolic reprogramming predominantly in macrophages rather than in renal tubular epithelial cells. This reprogramming is characterized by impaired fatty acid β-oxidation and enhanced glycolysis, which may contribute to TAC-induced kidney injury, whereas KYN can partially restore this metabolic imbalance.

### 3.5 Fatty acid oxidation deficiency inhibits M2 polarization and promotes renal inflammation

**FIGURE 6:**
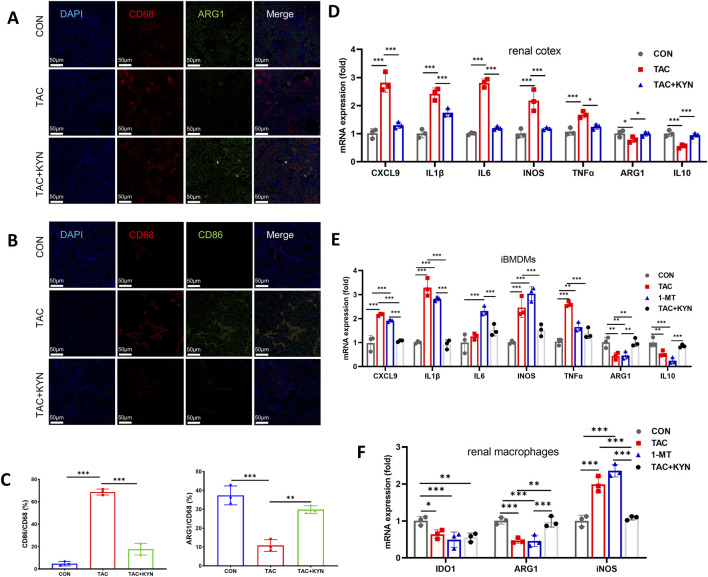
Indicators of macrophage polarization and proinflammatory cytokine expression. **(A)** Double immunofluorescence staining of CD68 (red) and ARG1 (green) in the renal cortex. **(B)** Double immunofluorescence staining of CD68 (red) and CD86 (green) in the renal cortex. **(C)** Quantification of CD86^+^/CD68^+^ (left) and ARG1^+^/CD68^+^ (right) double-positive cells (n = 3). **(D)** mRNA expression levels of M1-and M2-associated genes (CXCL9, IL-1β, IL-6, iNOS, TNF-α, ARG1, IL-10) in the renal cortex (n = 3). **(E)** mRNA expression levels of M1-and M2-associated genes in iBMDMs (n = 3). **(F)** mRNA expression levels of IDO1, ARG1, and iNOS in primary renal macrophages under indicated treatments (n = 3). Data are presented as mean ± SD. Statistical analyses were performed using one-way ANOVA followed by Tukey’s multiple comparisons test. *P < 0.05, **P < 0.01, ***P < 0.001.

Previous studies have shown that M1 macrophages primarily rely on glycolysis for energy production, whereas M2 macrophages depend mainly on FAO and oxidative phosphorylation OXPHOS (Cervenka et al., 2017; Zhai et al., 2024; Wang et al., 2023). To further explore whether TAC-induced metabolic disturbances affect macrophage polarization, we assessed the expression of M1 markers CD86/CD68 and M2 markers ARG1/CD68 in renal tissue by immunofluorescence staining (Figures 6A,B). The results showed that TAC markedly increased the overall presence of renal macrophages, increased the proportion of M1 macrophages, and reduced the M2 population. KYN supplementation effectively reversed this phenotypic shift, indicating its capacity to counteract TAC-induced proinflammatory polarization and restore anti-inflammatory macrophage identity (Figure 6C).

Additionally, we measured the expression of M1-and M2-associated cytokines in both mouse kidneys and iBMDMs *in vivo* and *in vitro*. RT-qPCR results showed that both TAC and the specific IDO1 inhibitor 1-MT significantly upregulated proinflammatory genes such as CXCL9, IL-1β, IL-6, TNFα, and inducible nitric oxide synthase (iNOS), while downregulating anti-inflammatory genes including IL-10 and ARG1. KYN treatment efficiently normalized these dysregulated transcriptional profiles (Figures 6D,E). Taken together, these findings demonstrate that IDO1 inhibition not only reshapes macrophage energy metabolism but also drives a shift toward the proinflammatory M1 phenotype while impairing the maintenance of the anti-inflammatory M2 phenotype.

To further validate these findings in primary macrophages, we examined gene expression in primary renal macrophages isolated from mouse kidneys. TAC treatment significantly reduced Ido1 mRNA expression in renal macrophages, consistent with regulation at the transcript level. In parallel, Arg1 expression was decreased, whereas Nos2 expression was increased. Similar trends were observed with 1-MT treatment, and KYN supplementation partially restored polarization-associated gene expression (Figure 6F).

### 3.6 Activation of PPARα restores fatty acid oxidation and prevents TAC-induced nephrotoxicity

**FIGURE 7:**
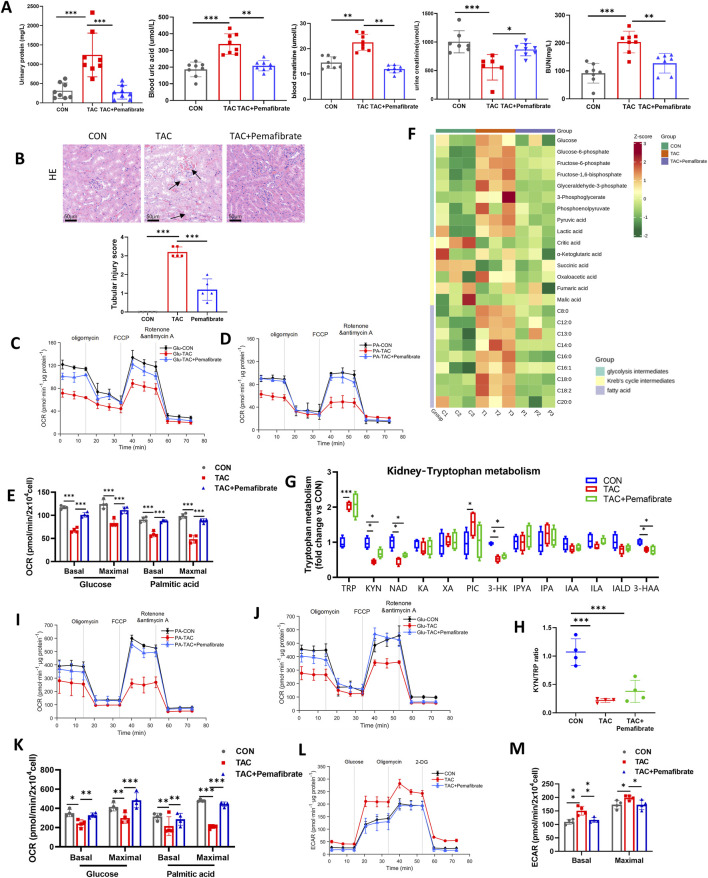
Indicators of renal function, energy metabolism, and Trp metabolism following PPARα agonist Pemafibrate intervention. **(A)** Biochemical indicators of kidney injury in mice, including urinary protein, serum creatinine, urinary creatinine, uric acid, and blood urea nitrogen levels (n = 8). **(B)** Representative H&E staining of kidney sections and corresponding tubular injury scores (n = 5). Arrows highlight tubular injury features. Scale bar = 50 μm. **(C,D)** Seahorse analysis of mitochondrial OCR in renal cortical cells under glucose **(C)** or palmitate **(D)** substrate conditions (n = 4). **(E)** Quantification of basal and maximal respiration in renal cortical cells corresponding to panels **(C,D)** (n = 4). **(F)** Heatmap showing the abundance of glycolysis intermediates, TCA cycle metabolites, and fatty acids in the renal cortex (n = 5). **(G)** Relative abundance of Trp metabolism–related metabolites in the renal cortex (n = 5). **(H)** Quantification of the KYN/Trp ratio in the renal cortex (n = 5). **(I,J)** Seahorse analysis showing mitochondrial OCR in iBMDMs under glucose **(I)** or palmitate **(J)** substrate conditions (n = 4). **(K)** Quantification of basal and maximal glycolytic capacity in iBMDMs corresponding to panel L (n = 4). **(L)** Seahorse analysis showing extracellular acidification rate (ECAR) in iBMDMs (n = 4). **(M)** Quantification of basal and maximal ECAR values in iBMDMs corresponding to panel L (n = 4). Data are presented as mean ± SD. Statistical analyses were performed using one-way ANOVA followed by Tukey’s multiple comparisons test.*P < 0.05, **P < 0.01, ***P < 0.001.

PPARα is highly expressed in the kidney and serves as a key transcription factor regulating fatty acid uptake, transport, and β-oxidation. To clarify the role of FAO impairment in TAC-induced nephrotoxicity, and to distinguish FAO-related effects from those caused by reduced NAD^+^ levels, we administered the PPARα-specific agonist Pemafibrate for intervention. Pemafibrate is a highly selective PPARα modulator that enhances fatty acid oxidation and improves lipid metabolism, and is clinically used for hypertriglyceridemia (Aomura et al., 2021; Horinouchi et al., 2023; Imai and Imai, 2022). *In vivo*, Pemafibrate significantly improved TAC-induced renal dysfunction, as evidenced by reduced levels of urinary protein, serum creatinine, and uric acid, along with restored urinary creatinine excretion (Figure 7A). Histological assessment further confirmed its protective effect, showing alleviation of tubular epithelial injury, tubular dilation, cast formation, inflammatory cell infiltration, and mesangial cell proliferation (Figure 7B).

Metabolomic and lipidomic profiling of iBMDMs revealed that PPARα activation markedly reversed TAC-induced accumulation of glycolytic intermediates and medium-to long-chain free fatty acids (Figure 7F). However, targeted Trp metabolomics showed that Pemafibrate failed to rescue TAC-induced alterations in Trp metabolism, including elevated Trp and decreased levels of KYN and NAD^+^, as well as reductions in downstream metabolites such as 3-HK and 3-HAA (Figure 7G). Moreover, IDO1 activity, as indicated by the KYN/Trp ratio, was not restored by Pemafibrate (Figure 7H). Mitochondrial respiration analysis of renal cortical cells showed that Pemafibrate significantly increased both basal and maximal oxygen consumption rate under glucose and palmitate substrate conditions following TAC treatment. The improvement was more pronounced under palmitate-driven respiration, suggesting that PPARα activation restores FAO and mitochondrial function to exert renoprotective effects (Figures 7C–E). In iBMDMs, the OCR pattern was consistent with *in vivo* findings (Figures 7I–K). In addition, ECAR measurements in iBMDMs demonstrated that PPARα activation also reversed TAC-induced glycolytic elevation (Figures 7L,M).

Taken together, these results indicate that even in the absence of NAD^+^ supplementation, restoring fatty acid β-oxidation alone is sufficient to alleviate TAC-induced kidney injury and macrophage metabolic reprogramming, underscoring the major contribution of FAO impairment to TAC-associated immunometabolic toxicity. Furthermore, the protective effect of KYN is primarily mediated through replenishment of NAD^+^ via the *de novo* pathway and enhancement of FAO, rather than through other NAD^+^-dependent mechanisms such as protein deacetylation or PARP regulation.

## 4 Discussion

In this study, we combined both *in vivo* and *in vitro* models to investigate the role of macrophages in the mechanism of TAC-induced nephrotoxicity. We identified that the Trp pathway, particularly the IDO1-mediated Trp-KYN pathway, was selectively suppressed by TAC. *In vivo*, TAC induced macrophage metabolic reprogramming by targeting IDO1targeted renal IDO1 activity, reducing *de novo* NAD^+^ synthesis and thereby inducing metabolic reprogramming in the kidney, including the accumulation of medium- and long-chain fatty acids and acylcarnitines, as well as enhanced glycolysis. This was accompanied by macrophage infiltration in the kidney, with a shift toward reduced M2 and increased M1 polarization. Exogenous supplementation with KYN reversed the decline in *de novo* NAD^+^ and corrected macrophage polarization, thereby alleviating renal injury. Moreover, restoring FAO through direct activation of PPARα with Pemafibrate improved renal metabolic function and alleviated tissue injury, indicating that enhancing FAO provides renal protection independently of Trp metabolism. *In vitro* metabolic profiling of HEK293T and iBMDMs revealed that iBMDMs, but not HEK293T cells, showed consistent metabolic changes with those observed *in vivo*, characterized by fatty acid accumulation and enhanced glycolysis following TAC or IDO1 inhibitor 1-MT treatment. These findings suggest that TAC induced macrophage metabolic reprogramming by targeting IDO1, thereby triggering a shift in polarization. Collectively, our data indicate that restoring FAO in renal macrophages by replenishing *de novo* NAD^+^ may offer a novel therapeutic strategy for TAC-induced kidney injury.

Previous studies on TAC-induced nephrotoxicity have primarily focused on its direct toxic effects on renal tubular epithelial cells, particularly via oxidative stress. For example, both coenzyme Q10 supplementation and ginsenoside-induced activation of the Klotho-PI3K/Akt/FoxO3a pathway have been shown to alleviate TAC-induced oxidative stress in tubular cells (Yu et al., 2019; Lim et al., 2019b; Liu et al., 2022). In addition, ROS overproduction and PARP activation have been implicated in NAD^+^ depletion and energy failure, linking calcineurin inhibition to metabolic collapse and fibrotic progression. The role of immune cells, especially macrophages, in TAC-induced tissue damage has gained increasing attention. TAC has been reported to inhibit M2 polarization by targeting Janus kinase 2 (JAK2) and suppressing the JAK2/STAT3 pathway (Luo et al., 2019). However, most studies have concentrated on TAC-induced macrophage infiltration and the enhanced secretion of proinflammatory cytokines, while little is known about the contribution of macrophage metabolic states to TAC nephrotoxicity (Wang et al., 2021; Kidokoro et al., 2012). Our findings suggest that IDO1-dependent impairment of *de novo* NAD^+^ synthesis may intersect with these classical injury pathways by reducing NAD^+^ availability, thereby aggravating mitochondrial dysfunction and driving macrophage metabolic reprogramming toward a proinflammatory state. Thus, rather than replacing established mechanisms, IDO1-mediated metabolic dysregulation may act as an upstream immunometabolic amplifier within the broader framework of TAC-induced renal injury.

NAD^+^ plays essential roles in cellular health, stress responses, and renal homeostasis. It has been reported that TAC induces reactive oxygen species (ROS) production, which activates poly (ADP-ribose) polymerase (PARP), leading to NAD^+^ depletion, mitochondrial dysfunction, and renal fibrosis (Kern et al., 2014; Kim et al., 2023). In addition, plasma metabolomics has implicated impairment of the NAD^+^ salvage pathway as a potential contributor to TAC-induced kidney injury (Nishida et al., 2025). Most current strategies for NAD^+^ supplementation rely on the nicotinamide salvage pathway through NAM, which requires the activity of nicotinamide phosphoribosyltransferase (NAMPT) (Zhang et al., 2025). However, NAMPT activity is often suppressed under inflammatory conditions. Another route is the Preiss–Handler pathway fueled by NA, but its rate-limiting enzyme NAPRT is likewise downregulated in inflammation. Increasing evidence highlights the importance of *de novo* NAD^+^ synthesis, previously regarded as a minor route compared to nicotinamide recycling. In kidney disease, enzymes in the *de novo* pathway such as quinolinate phosphoribosyltransferase (qPRT), 3-hydroxyanthranilic acid dioxygenase (HAAO), and aminocarboxymuconate-semialdehyde decarboxylase (ACMSD) are being recognized as potential therapeutic targets (Wang et al., 2022; Poyan et al., 2018; Minhas et al., 2019). Moreover, recent studies have highlighted *de novo* NAD^+^ synthesis in macrophages as essential for maintaining OXPHOS and immune homeostasis. For instance, restoring *de novo* NAD^+^ levels by targeting qPRT decreases glycolytic intermediates while increasing TCA cycle metabolites in macrophages (Minhas et al., 2019). Together, these findings underscore the metabolic and immunological significance of the *de novo* NAD^+^ biosynthetic pathway and support its potential involvement in TAC-induced nephrotoxicity.

Additionally, IDO1 activity has been proposed as a biomarker for early diagnosis of CKD (Minhas et al., 2024). In this study, we systematically confirmed—through gene expression and metabolite analyses at both tissue and cellular levels—that TAC was associated with impaired IDO1 activity and suppression of the KP, accompanied by reduced *de novo* NAD^+^ synthesis. Supplementing with KYN as a precursor for *de novo* NAD^+^ synthesis bypassed NAMPT dependency and effectively restored NAD^+^ content and FAO capacity in the kidney. Furthermore, inhibition of IDO1 has also been shown to enhance glycolysis and lactate production in astrocytes, highlighting the broader metabolic influence of the KP and NAD^+^ biosynthetic pathways (Huang et al., 2016). In line with this metabolic framework, an independent liver-fibrosis study showed that corilagin suppresses IDO1 and repolarizes macrophages from M2 to M1, thereby alleviating fibrosis. In liver fibrosis, where M2/pro-fibrotic polarization predominates, reducing IDO1 helps disrupt the pro-fibrotic inertia and mitigates disease progression. By contrast, in TAC-induced nephrotoxicity, IDO1 inhibition exacerbates NAD^+^ depletion and FAO impairment, thereby driving M1 polarization and amplifying inflammation and tissue injury. Despite these directional differences, both contexts highlight the central role of the IDO1-mediated regulation of the KP in controlling macrophage metabolic polarization (Chen et al., 2024).

PPARα is a nuclear receptor abundantly expressed in the kidney that regulates fatty acid uptake and mitochondrial β-oxidation. In this study, pemafibrate improved renal function and restored OCR particularly under palmitate-driven respiration, highlighting its capacity to directly rescue FAO. Pemafibrate is a novel lipid-lowering drug, and its clinical application is currently limited to the treatment of dyslipidemia, with no established therapeutic role in kidney disease. Moreover, as a nuclear receptor, PPARα regulates a wide range of downstream proteins, and the precise mechanisms underlying its renal effects remain incompletely understood. By contrast, IDO1 represents a more defined and targetable molecule. In this study, we clearly demonstrated the mechanistic relationship between TAC-induced kidney injury and IDO1 suppression. Therefore, although PPARα agonists may confer broad metabolic benefits, targeting IDO1 may represent a mechanistically defined therapeutic direction.

At the metabolic level, TAC-induced macrophage reprogramming was characterized by the accumulation of medium- and long-chain fatty acids and acylcarnitines together with enhanced glycolysis, whereas most TCA cycle intermediates remained stable except for a selective reduction in succinate. Given that fatty acid β-oxidation requires adequate NAD^+^ supply to sustain multiple dehydrogenase reactions, suppression of IDO1 with consequent impairment of *de novo* NAD^+^ synthesis provides a mechanistically plausible upstream event leading to FAO disruption and compensatory glycolytic activation. Consistently, pharmacological inhibition of IDO1 recapitulated the FAO-related metabolic phenotype, and KYN supplementation restored NAD^+^ levels while reversing lipid accumulation and glycolytic enhancement.

Several limitations should be acknowledged. First, this study was conducted exclusively in male C57BL/6J mice, and potential sex- or strain-specific differences were not evaluated. In addition, the long-term effects of TAC exposure and metabolic intervention were not systematically assessed, limiting conclusions regarding chronic outcomes and safety. In the present study, KYN supplementation was applied under TAC-induced IDO1 suppression to restore *de novo* NAD^+^ synthesis. However, recent studies have reported that exogenous KYN may impair renal structure and function under certain experimental conditions (Krupa et al., 2022; Irsik et al., 2024). Therefore, its independent renal effects and long-term systemic safety require further systematic evaluation.

At the mechanistic level, succinate was selectively reduced following TAC treatment; however, this decrease was neither restored by KYN supplementation nor reproduced by pharmacological IDO1 inhibition, suggesting that this specific TCA alteration is unlikely to be mediated by IDO1 suppression and subsequent *de novo* NAD^+^ depletion. Instead, it may reflect additional mitochondrial effects of TAC that require further investigation. Given that the classical “M1 = glycolysis” and “M2 = FAO” dichotomy is increasingly questioned (Liu et al., 2022), our data support a network-level metabolic disruption rather than a simple polarization shift. Given that the accumulation of medium- and long-chain fatty acids and acylcarnitines is typically indicative of limitations in β-oxidation, our data suggest that TAC may impair fatty acid degradation. However, identifying the precise metabolic node affected by TAC—whether fatty acid uptake, the carnitine shuttle, or transcriptional regulation of β-oxidation pathways such as PPARα or PGC1α—will require further investigation. Defining these metabolic disruptions will be essential for understanding TAC’s immunometabolic toxicity and for developing more targeted therapeutic strategies. Finally, although biochemical dependency supports a hierarchical model in which IDO1 suppression and subsequent *de novo* NAD^+^ depletion precede metabolic reprogramming, a formal time-course analysis was not performed, and future temporal studies will be necessary to establish the sequential relationship more rigorously.

In summary, TAC impairs IDO1-dependent regulation of the KP and *de novo* NAD^+^ synthesis, thereby contributing to macrophage metabolic reprogramming and renal inflammation. Restoration of *de novo* NAD^+^ synthesis or enhancement of fatty acid oxidation (FAO) attenuated these alterations, highlighting macrophage metabolism as a viable therapeutic target in TAC-induced nephrotoxicity.

## Data Availability

The raw data supporting the conclusions of this article will be made available by the authors, without undue reservation.
