# Supplementary material for: Indoleamine 2,3-dioxygenase-regulated macrophages metabolic reprogramming rescues tacrolimus-induced nephrotoxicity
Source: Front Pharmacol. 2026 Mar 18;17:1784153. doi: 10.3389/fphar.2026.1784153 (PMC13038910; doi:10.3389/fphar.2026.1784153)
Supplement: Supplementary file 1 [file Table1.docx]

Table S1. Primer sequences used for RT-qPCR analysis.

| **Gene** | **Forward (F) Primer** | **Reverse (R) Primer** |
| --- | --- | --- |
| **IL-1β** | GCAACTGTTCCTGAACTCAACT | ATCTTTTGGGGTCCGTCAACT |
| **IL-6** | TAGTCCTTCCTACCCCAATTTCC | TTGGTCCTTAGCCACTCCTTC |
| **CXCL9** | GGAGTTCGAGGAACCTGATGT | GGGATTTGTAGTGGATCGTGC |
| **iNOS** | CACCTTGGAGAGGAGAGAACTAC | GAGCAGAAAGGCGCAGAACTGA |
| **TNFα** | CCTGTAGCCCACGTCGTAG | GGGAGTAGACAAGGTACAACCC |
| **IDO1** | CAAATGCAAGACGGAACACT | TGCCTTTCCAGCAGACACAA |
| **ARG1** | AGCTCTGGGAATCTGCATGG | ATGTACACGATGTCTTTGGCAGATA |
| **IL-10** | GCTCTTACTGACTGGCATGAG | CGCAGCTCTAGGAGCATGTG |
| **CPT1A** | TGGGCATCATCACTGGTTGTGTT | TGGGCATCATCACTGGTTGTGTT |
| **CPT2** | TGGGCACCTGAGGTCCTGT | GCCGCCAGTGCAAAAGCATGT |
| **LCAD** | CTGGTGATTGTTGGTGCGTGT | CTGGTGATTGTTGGTGCGTGT |
| **MCAD** | AGAGGAGATTTACCCGTGGGC | TACGCCAACTCTTCGGTAACTAAAC |
| **β-Actin** | CATTGCTGACAGGATGCAGAAGG | TGCTGGAAGGTGGACAGTGAGG |

**Note:** The forward (F) and reverse (R) primer sequences for each target gene were used in RT-qPCR analysis. All primers were synthesized by Tsingke Biotechnology, Beijing, China.
